# Market access agreements for pharmaceuticals in Europe: diversity of approaches and underlying concepts

**DOI:** 10.1186/1472-6963-11-259

**Published:** 2011-10-08

**Authors:** Szymon Jarosławski, Mondher Toumi

**Affiliations:** 1 Creativ-Ceutical, 215, rue du Faubourg St-Honoré, 75008 Paris - France; 2 Institute of Bioinformatics and Applied Biotechnology, Biotech Park, Electronics City Phase I, 560100 Bangalore - India; 3 University Claude Bernard Lyon I, UFR d'Odontologie, 11, rue Guillaume Paradin, 69372 LYON CEDEX 08 - France

## Abstract

**Background:**

Market Access Agreements (MAA) between pharmaceutical industry and health care payers have been proliferating in Europe in the last years. MAA can be simple discounts from the list price or very sophisticated schemes with inarguably high administrative burden.

**Discussion:**

We distinguished and defined from the health care payer perspective three kinds of MAA: Commercial Agreements (CA), Payment for Performance Agreements (P4P) and Coverage with Evidence Development (CED). Apart from CA, the agreements assumed collection and analysis of real-life health outcomes data, either from a cohort of patients (CED) or on per patient basis (P4P). We argue that while P4P aim at reducing drug cost to payers without a systematic approach to addressing uncertainty about drugs' value, CED were implemented provisionally to reduce payer's uncertainty about value of a medicine within a defined time period.

**Summary:**

We are of opinion that while CA and P4P have a potential to reduce payers' expenditure on costly drugs while maintaining a high list price, CED address initial uncertainty related to assessing the real-life value of new drugs and enable a final HTA recommendation or reimbursement and pricing decisions. Further, we suggest that real cost to health care payers of drugs in CA and P4P should be made publicly available in a systematic manner, to avoid a perverse impact of these MAA types on the international reference pricing system.

## Background

Achieving broad market access for new pharmaceutical products has become critical for their manufacturers. This is because high price itself might not directly translate into high revenue: sales volume might be reduced drastically if an expensive pharmaceutical product falls under scrutiny of health care payers and is refused reimbursement or is not recommended for use by health technology assessment (HTA) process. While cost-effectiveness analysis is primarily used to aid decision making in the UK or Sweden, other countries (such as France or Germany) might refer to "economic resource use" rather than to specific type of analysis [1].

In practice, costly drugs are subject to market access negotiation, i.e. finding a compromise between health care payers and the industry on the drug's price and reimbursement status, HTA recommendation (for specific populations of patients) and/or formulary listing. The outcome of this process can thus be called a Market Access Agreement (MAA). Clearly, an obvious alternative to many often sophisticated MAA would be to bring the drug's list price closer to a level of a currently used treatment option - a solution deemed unacceptable by the industry, particularly in disease areas where many generic therapeutic options are available. Even more so because out of 27 EU member states, 24 use international referencing to set prices of new drugs [2]. Consequently, lowering the list price in one country might result in lower prices in countries which will later use it for reference. From this perspective, the industry may choose to launch their drugs sequentially, starting from countries where they are likely to achieve a high list price [2].

In a recent review paper European payers distinguished two types of agreements: financially-based and payment-for-performance [3]. We felt that there had been little debate among the European health care payers on what were their motivations to implement various types of schemes in their respective countries and what impact it might have on prices in peer EU member states. Here, we present an opinion paper based on a succinct review of MAA other than price-volume agreements and market caps. We use a perspective of the health care payer and we attempt to classify MAA according to characteristics such as collection and analysis of real-life patient health outcomes and the duration of the agreements (provisional or permanent).

## Methodology

The aim of this debate paper is to contribute to the general debate on Market Access Schemes based on information which is published publicly by relevant national health care payers in Europe. We used a review paper written by public health care payers to identify European Union countries where MAA were implemented (UK, France, Italy, and Denmark) [3]. This paper was chosen because it was a literature review supplemented by unpublished or "grey literature" references known to 16 European co-authors, mostly health authority and health insurance personnel evaluating and implementing such schemes [3]. For the purpose of this paper we excluded price-volume agreements and market caps, as well as schemes that were direct-to-customer awareness/marketing campaigns rather than MAA with health authorities (i.e. the "140-90 initiative" for valsartan from Novartis [4] and Bayer' scheme on vardenafil [5] in Denmark). Additionally, we identified through personal communication that MAA were launched in Sweden [6]. Therefore, we searched websites of national HTA and reimbursement authorities in UK (England and Wales) (National Institute for Health and Clinical Excellence (NICE) and Department of Health (DoH), Italy (Agenzia Italiana del Farmaco (AIFA)), France (Haute Authorite de Sante (HAS)), and Sweden (The Dental and Pharmaceutical Benefits Agency (TLV)) for documents containing the following keywords: "risk-sharing", "patient access scheme", "coverage with evidence development', "conditional coverage/reimbursement", "outcome guarantee", registry, "observational study" or "payment/pay by performance". Non-English documents and websites were translated by native speakers. For retrieved documents, we sought to identify additional information in general Web resources and we provide reference for each. All searches were performed between September and December 2010. Because this paper is a "debate communication" we did not use the standard literature review methodology and searches were performed by one author. However, both authors were independently involved in analysis and abstraction of data.

## Discussion

### Review of Market Access Agreements

Table 1 summarizes MAA which we identified in documentation published by European health care payers on their websites. Below, we analyse them with respect to the collection and analysis of real-life patient health outcomes and the duration of the agreements (provisional or permanent).

**Table 1:** Market Access Agreements identified on websites of European health care payers and their classification according to the typology proposed by authors.

Market Access Agreement	Analysis of health outcomes data from a cohort or on per patient basis	Provisional agreement or permanent risk-shifting	Type of MAA
Two MAA for levodopa/carbidopa and rimonabant in Sweden [6-10]	cohort	provisional	CED

12 MAA for oncology drugs in Italy [13]	cohort and per patient	provisional	P4P or CA

One MAA for three Alzeimer's Disease drugs in Italy [3,16]	cohort and per patient	provisional	CED

Three MAA for angina pectoris and resistant type 2 diabetes drugs in Italy [14,17-19]	cohort	provisional	CED

11 MAA developed as a part of NICE appraisal process [21-36]	per patient**	permanent**	P4P or CA; (CED**)

One MAA for Multiple Sclerosis drugs in UK [3,37]	cohort	provisional	CED

Two MAA for type 2 diabetes drugs in France [12]	cohort	provisional	CED*

The MAA for RisperdalConsta LR in schizophrenia in France [11,12]	cohort	provisional	CED*

For review of the schemes see [3,7,10,38].

*- unfavourable CED results assumed reduction of list price of the drug and is some cases also a rebate of money for sold packages

**-one PAS assumed price reduction in case of unfavourable results of an ongoing clinical trial versus a comparator drug

Swedish TLV, which uses cost-effectiveness analysis to inform its decision making, does not recommend for reimbursement drugs which showed uncertain or high (above a certain disease-specific threshold) ICER value. Levodopa/carbidopa's (duodopa(R)) path to final pricing and reimbursement in Sweden went through a temporary (five-year long) MAA which aimed at generating real-life evidence that would allow to reduce the value of ICER and uncertainty around it [6-8]. Briefly, while the product was granted provisional reimbursement at a premium price at the time of initial manufacturer submission, additional cost-effectiveness analyses and follow-up studies enabled TLV to make a final positive reimbursement decision at the premium price. Another example was a MAA for rimonabant in the treatment of obesity, which was also granted an interim reimbursement status for a period of two years. The final TLV decision was conditional on additional data showing long-term effects and cost-effectiveness of the drug in real-world practice [7,9,10].

We identified two requests of the French Haute Authorite de Sante (HAS) of real-life comparative studies which were expected to reduce uncertainty about drugs' real-life performance and enable final pricing. In 2005, the French pricing body Comité économique des produits de santé (CEPS) asked the manufacturer of the injectable antipsychotic risperidone (RisperdalConsta LP) to perform a one-year study that should evidence reduction in a rate of hospitalizations for patients treated with this drug as compared to other antipsychotics (to be designed under supervision of Ministry of Health) [11,12]. This ESCROW MAA assumed that while the drug will be granted a premium list price (approximately 15-fold premium versus generic injectable LP antipsychotics and almost 60% versus oral risperidone from Janssen-Cilag), the company would receive payment based on the price of cheaper comparators and the difference would be deposited as public funds in Caisse des Dépôts et Consignations until results from the study are available. Shall the results evidence reduction in hospitalization rate, money would be transferred to the company. Otherwise, social security services would receive the funds. Another MAA requested by CEPS and the French HTA agency Commission de Transparence was a real-life use study for glitazones (pioglitazone and rosiglitazone) in type 2 diabetes. This 2 year observational study was proposed in 2004 and was expected to develop evidence that would support or oppose manufacturer's claim of a superior real-life efficacy (time to introduction of an add-on therapy) than previously observed in clinical trials.

We identified 19 various MAAs in Italy, but there were no detailed technology appraisals available on AIFA's website. Overall, there were 12 MAAs for oncology drugs: erlotinib (2006), sunitinib (2006), sorafenib (in advanced renal cell carcinoma in 2006 and liver cancer in 2008), dasatinib (2007), bevacizumab (2008), lenalidomide (2008), temsirolimus (2008), bortezomib (2009), cetuximab (2009), lapatinib (2009), panitumumab (2009) and trabectedin (2009) [13]. The MAA involved fixed discounts (from the list price) and/or pay-backs for non-responding patients (100% or 50% of the drug's cost, all on per patient basis). While safety and efficacy of the drugs were monitored in patient registries, it is noteworthy that those MAA did not seek to answer uncertainty about a clearly specified health outcome and data collection in the registries was not systematic with a high potential for various bias [14]. E.g. it was estimated that in some regions of the country only 50% of patients were covered by the registries and no process had been put in place to ensure an unbiased selection of patients which might have led to doctors feeding records only for patients for which less administrative burden was expected [15].

In contrast, the CRONOS project launched by AIFA to evaluate real-life effectiveness of Alzheimer's Disease drugs (donepezil, rivastigmine and galantamine) assumed collection and analysis of well-defined health outcomes from a cohort of patients. It was carried out in a nationally representative sample of patients with AD over a period of two years and the public insurer reimbursed medicines only in patients who responded at four months of treatment (while the cost for non-responders was covered by manufacturers) [3,16].

Other examples of MAA which we identified in Italy were for drugs indicated in chronic angina pectoris (ivabradine) [17] and type 2 diabetes mellitus (sitagliptin, vilagliptin and exenatide) [14,18]. These provisional MAA were set up to monitor real-life use, collect epidemiologic data as well as new efficacy and safety data for re-assesment of price and/or reimbursement conditions for the medicines by the Italian agency. The schemes were run with a restriction of treatment initiation to specialist centres, monitoring of clinical practice, adverse events and withdrawals due to treatment failure [19].

In contrast to the above HTA agencies, NICE publishes drug appraisal documents which include detailed information on any MAA in place. At the time of writing this article, 11 Patient Access Schemes (PAS) for ten medicines were developed as a part of NICE appraisal process [20] and all were financially-based agreements, according to the DoH's definition [21-23]. These medicines were: erlotinib in non small cell lung cancer [24], lenalidomide in multiple myeloma [25], ranibizumab in acute wet macular degeneration [26], trabectedin in advanced soft tissue sarcoma [27], gefitinib in non small cell lung cancer [28], sunitinib in advanced and/or metastatic renal cell cancer [29] and in gastrointestinal stromal tumours [30], cetuximab in colorectal cancer [31], certolizumab in rheumatoid arthritis [32], ustekinumab in psoriasis [33], bortezomib in multiple myeloma [34], azacitidine in myelodysplastic syndromes [35] and pazopanib in first line advanced renal cell cancer [36]. All PAS did not require collection of data from cohorts of patients. In the case of pazopanib, the scheme assumed a value rebate and subsequent price reduction in the event that the drug fails to prove its non-inferiority in an ongoing head-to-head clinical trial versus one of the comparators (sunitinib). In the remaining cases, PAS did not envisage scheduled price revision (in UK drug list prices are notified by manufacturers to the MoH) or any other final decision making with respect to these drugs. Nevertheless, we note that the 2009 Pharmaceutical Price Regulation Scheme stipulates that when designing PAS, clarity is required on the exact duration of any agreement and the circumstances in which it might be terminated [22] and that NICE schedules regular revisions of its drug appraisals so that the HTA recommendations are subject to change.

Apart from the above MAA developed as a part of NICE drug appraisal process, the UK's DoH launched in 2002 a MAA for β-interferons and glatiramer in the treatment of multiple sclerosis (MS). This experimental scheme was agreed with four manufacturers of disease-modifying MS drugs and assumed collection and analysis of health outcomes (Expanded Disability Status Scale) data from a cohort of approximately 10,000 patients, followed for over 10 years. Briefly, the cost of drugs incurred by the NHS would be reduced if a new ICER estimate calculated over an envisaged 20-year horizon was above £35,000/QALY [3,37].

Interestingly, there was no synergy between construction of UK's and Italian schemes for the same drugs, e.g. bortezomib (in multiple myeloma) which was available in UK within a payment-for-performance (per patient) agreement, in Italy was reimbursed within a MAA that featured a fixed cost-share (per patient) and a discount (from the list price).

### Classification of Market Access Agreements Revisited

Based on the above review of the MAA we propose that the approach to the collection and analysis of patient health outcomes data by payers (none, cohort or per patient) and the timeframe of the agreement (provisional or permanent) can be used to classify them into three major categories: Commercial Agreements (CA), Payment for Performance Agreements (P4P) and Coverage with Evidence Development (CED). This classification is meant to help the reader quickly identify the underlying concepts of various MAA they may come across in their practice. Table 2 attempts to define these types of MAA by summarizing their major features and by giving relevant examples.

**Table 2:** Definitions and examples of the three types of Market Access Agreements

MAA Category	Commercial Agreement	Payment for Performance Agreement	Coverage with Evidence Development
Contract type	• Discount-based contract	• Permanent risk-shifting agreement (outcomes guarantee/insurance) applied on a per-patient basis	• Provisional agreement until new, clearly specified evidence develops from a cohort of patients

Collection and analysis of patient health outcomes data by the payer	• None	• Per patient	• Cohort of patients

Timeframe of the MAA	• Permanent/not linked to final decision-making	• Permanent/not linked to final decision-making based on new robust evidence	• Temporary/provisional until new evidence allows making a final decision

Underlying concept (payer perspective)	• Reducing pharmaceutical expenditure	• Avoiding inefficient expenditure on treating patients who do not respond to a drug and who cannot be identified *ex ante *(by permanently linking the payment to drug's performance in individual patients)	• Reducing uncertainty about drug's real-life effectiveness (by linking a final HTA, reimbursement and/or pricing decision to drug's performance in a cohort of patients, during a defined test period)

Examples	• Flat price per patient (regardless of the number of doses administered) • Cost Sharing • Rebate • Discount	• Payment for performance • Pay-back for non performance	• Temporary coverage on a condition that new evidence reduces uncertainty about a pre-specified health outcome: - Real-life effectiveness - Higher efficacy in a pre-specified subpopulation of patients - Long-term effect - Improved patient's adherence - Reduction of use of health care resources (e.g. hospitalization)

CA typically aim at reduction of expenditure on costly drugs for the health care payer, without collecting and analyzing real-life health outcomes data from patients. While in principle they can be renegotiated, they are permanent agreements in a sense that they do not assume a future final reimbursement decision in light of new data on pre-specified health outcomes from a well-designed study.

While CED agreements are provisional by nature and involve running a health outcomes study on a cohort of patients, P4P are managed on a per patient basis without attempting to answer uncertainty about drug's cost- or clinical-effectiveness. In other words, while CED always leads to a scheduled reassessment of the drug's (cost-) effectiveness, price revision and to regular reimbursement status, P4P is set to pay only for patients who achieve a pre-specified response to a drug. While P4P must involve defining individual patient's response, it does not have the potential to deliver high-quality data on drug's actual (cost-) effectiveness and does not lead to a more evidence-based reimbursement decision or HTA recommendation.

We note that CED itself can be a costly process as it involves purchasing the medicine for patients. Therefore, it is not surprising that e.g. Italian AIFA chose to pay only for responding patients during the CRONOS CED. Nevertheless, it is clear that the main objective of the scheme was to develop new evidence on real-life health outcomes from a cohort of patients which would enable a final reimbursement decision and therefore it can be classified as CED.

Further, medicines in some P4P schemes in Italy were purchased with a discount from the list price. Since AIFA documentation did not refer to cost-effectiveness *per se*, the main objective of the schemes was to enable positive reimbursement recommendation on these drugs while ensuring a lower budgetary impact. Supposedly, the agency felt that the financial outlays would be too high even if only treatment of responding patients was financed and therefore sought to agree with the manufacturers on discounts from list prices of concerned drugs. Therefore, those schemes can be classified as P4P/CA

We show in the last column in Table 1 how the MAA which we reviewed here can be categorized according to this classification (for a detailed review of the schemes see [3,7,10,38])

### Performance and the future of MAA

Some academics argue that P4P or CED are implemented when the payer perceives high risk related to paying for a medicine (it is uncertain that financing a costly drug is good value for money), and the manufacturer is confident that the product has good efficacy (value) [39]. The payer's uncertainty is more pronounced in the case of newly launched medicines or old ones for which no post-marketing data exists. This is because drugs are licensed in the light of data from highly controlled clinical trials performed often on selected patient populations and payers are concerned about the drug's real-practice effectiveness in general patient population and/or about its impact on use of other resources in the health care system. From that perspective, the ultimate goal of P4P and CED for a payer should be therefore minimization of a possibility that it finances a technology that is not (cost-) effective in real-life use [40]. However, we believe that there is a substantial difference between attempting to reduce the financial outlays of purchasing a costly medicine because it is not considered value for money and seeking to develop clearly specified real life evidence to reduce payer's uncertainty about the value of the drug. As we discuss below, while P4P can merely improve payer's *acceptability *to finance a costly medicine, CED actually addresses the *uncertainty *related to the decision to finance a medicine.

In Sweden, the two CED discussed earlier enabled the payer to collect evidence that led to re-evaluation of the real-life cost-effectiveness of concerned drugs and final reimbursement and pricing decisions were delivered. Following these decisions, the drugs were financed without employing any further MAA.

In the case of French CED for RisperdalConsta LP, five years after the initial HAS ruling of minor improvement of clinical benefit (Amelioration du Service Medical Rendu (ASMR) IV), the requested study provided evidence that in a cohort of over 1600 patients followed for 1 year, patients treated with the concerned drug had a lower relative risk of hospitalization than those on other antipsychotics [11] and so the premium list price has been maintained. On the other hand, the observational study for rosiglitazone (AVANCE) largely repeated the efficacy data which had been shown in clinical trials and therefore did not support manufacturers' claims of a superior real-life efficacy [12,41]. As a consequence of this result the pricing committee CEPS cut the drug's price by 30%, requested rebates for the drug already purchased and altered the reimbursement level from 65% to 35%. However, no systematic information on the actual amounts of the rebates was published by HAS.

On the other hand, the experimental CED for MS drugs in UK was inconclusive at seven years from its launch. While access to the drugs varied across the UK and remained the lowest among peer countries [42], the evidence on the drugs' efficacy that was gathered from a cohort of over 5,500 patients did not reduce uncertainty surrounding the ICER to a level that would allow price revision [43].

While the design of Patient Access Schemes (PAS) developed as a part of NICE appraisal process addresses uncertainty about the ICER estimate from cost-effectiveness analysis [38], there have been issues with their real-life performance and savings brought to the NHS. A report from the British Oncology Pharmacy Association concluded that hospital pharmacies would rather not have to deal with PAS and would prefer a straight forward discount [44]. Further, data from 31 hospitals in the UK showed that, between 2007 and 2009, 47% of manufacturer pay-backs arising from sunitinib and bortezomib PAS were not recovered by Primary Care Trusts [45]. It is not surprising that the chair of NICE, Sir Michael Rawlins has said recently that "(...) a simple discount may eliminate the need to put in place complicated schemes that require substantial management input" [46]. He further suggested the initial discounts that could replace complex schemes should amount to about 30% of the list price. Nevertheless, it is arguably too early to judge the performance of those schemes. Further, a recent PAS for gefitinib is a sign that NICE and DoH continue to develop MAAs being a mix of P4P and CA, but give more consideration to prior assessment of their future administrative impact.

The Italian AIFA announced that following an analysis of patients' registries which were a part of P4P and CA agreements for expensive cancer drugs, it would reduce their list price by 30-40% in 2011 [47]. While scheduled price revision was assumed at the launch of these P4P schemes, they had not been designed to answer uncertainty about (cost-) effectiveness of the drugs and it is unlikely that the registry data had the sufficient quality to provide more robust estimates than those available at drugs' launch.

On the other hand, the Italian CED for AD drugs (CRONOS), provided new real-life effectiveness data and allowed AIFA to reimburse these medicines, with some restrictions with respect to diagnosis and continuation of treatment and prescription limited to specialist physicians [48].

The payers had a diversity of approaches towards how to sponsor provision of the drug for patients during its testing CED period. In France, drugs in one CED were financed by the payer at a premium price, but in case of unfavourable results the difference between the cost of a cheaper comparator and the premium price was returned to the payer. In Sweden, the drugs were financed at a premium price and in Italy at a premium price with payment limited to patients who responded to treatment (for the CRONOS MAA). In UK, the MS disease-modifying drugs were financed at a premium price, but the scheme assumed sliding reduction of the price as soon as unfavourable interim evidence from the CED becomes available.

Finally, as we noted earlier, almost all European national health care payers use list prices in peer countries to set drug prices. Clearly, CA and P4P obscure the real financial outlay of concerned drugs to the payer who agreed on them and the list price becomes meaningless for international referencing. This is also the case if drugs in CED are financed by the payer at a premium list price as the drug will likely be launched (and priced) in other countries during the CED period.

For that reason we are of opinion that the industry chooses to propose such schemes to payers because of their interest in maintaining high list prices. The payers agree because such MAA allow them to reduce financial outlays on costly medicines in their own countries. Unfortunately, as a "side effect" of such policy, prices in peer countries might be set with reference to the arguably unwarranted, "facial" list price. However, if the real drug's price (i.e. the financial outlay to the payer) becomes available for reference, the industry might lose their interest in this often sophisticated form of contracting and lean towards rethinking of their pricing strategies. This is currently not possible as details of many MAA are held under Commercial in Confidence agreements and also because real spending on drugs in MAA is not published by payers in a systematic manner. At the same time, patient registry data is not available to third parties for independent analysis.

## Summary

It appears from our discussion that well designed and transparent CED have proven to be a powerful tool which enables reduction of uncertainty about drug's real-life performance. The examples of CED developed in France, Italy and Sweden, suggest that reducing payer's uncertainty about drugs value is indeed feasible, shall this be the true motivation of payers. While CA and P4P have a potential to incur savings to individual national payers, care should be taken that the real cost of purchasing drugs in MAA, rather than their list price, is used for international reference pricing in Europe.

## Competing interests

The authors declare that they have no competing interests.

## Authors' contributions

SJ performed searches and analyses and drafted the manuscript. MT was at the origin of the original idea of the article and was involved in the interpretation of the data. All authors read and approved the final manuscript.

## Pre-publication history

The pre-publication history for this paper can be accessed here:

http://www.biomedcentral.com/1472-6963/11/259/prepub
